# Plasma Microbial Cell-Free DNA (CF-DNA) Next-Generation Sequencing in Diagnosing Intracranial Abscesses: Pathophysiology and a Scoping Review of the Literature

**DOI:** 10.7759/cureus.28172

**Published:** 2022-08-19

**Authors:** Bahadar S Srichawla

**Affiliations:** 1 Department of Neurology, UMass Chan Medical School, Worcester, USA

**Keywords:** fusobacterium nucelatum, rothia mucilaginosa, neuroinfectious disease, cell free dna, karius test, infectious disease, neurocritical care, neurology, intracranial abscess, cerebral abscess

## Abstract

Plasma microbial cell-free DNA (cf-DNA) from next-generation sequencing (NGS) provides improved sensitivity and specificity compared to standard microbial blood cultures. cf-DNA sequencing also has an improved turnaround time (TAT) and allows quicker commencement of antibiotics in life-threatening infections such as a brain abscess. Brain abscesses carry significant morbidity and mortality. Empiric treatment and management are critical in improving functional neurological outcomes. Reported here is the case of a severe central nervous system (CNS) infection with multiple ring-enhancing lesions seen throughout the cerebrum on magnetic resonance imaging (MRI). Standard microbial blood cultures were inconclusive and definitive identification of the pathogen was achieved through microbial cf-DNA NGS.

Brain abscesses develop in four distinct phases: early cerebritis, late cerebritis, early capsule formation, and late capsule formation. The pathogenesis of cerebral abscess involves direct parenchymal inflammation of the pathogen, the recruitment of inflammatory CNS cell types (microglia, inflammatory astrocytes, etc), and the chemotaxis of immune cells. cf-DNA is released into the bloodstream in response to pathogen opsonization and immune-mediated cell death. A scoping literature review includes cases of intracranial abscesses diagnosed via cf-DNA NGS.

## Introduction

Obtaining microbial cultures is a gold standard in diagnosing infections; however, many pathogens remain undetected with standard blood cultures. Cell-free DNA (cf-DNA) refers to all nonencapsulated DNA in the blood. cf-DNA is released into the blood after cell lysis, apoptosis, or necrosis. Plasma microbial cf-DNA (mcf-DNA) sequencing allows the detection of cf-DNA and offers a higher diagnostic yield by checking the presence of more than 1,200 pathogens [1]. The turnaround time (TAT) for mcf-DNA sequencing is notably faster than standard microbial blood cultures. The increased diagnostic yield and improved TAT provide significant clinical benefits not only in diagnosing severe systemic infections but also in determining the appropriate treatment strategy. 

Brain abscesses are often fatal if left untreated and can have a mortality rate of approximately 10% [2]. Reported here is a case of an intracranial abscess in a 34-year-old male presenting with seizures and multiple focal lesions on magnetic resonance imaging (MRI) of the brain. Standard microbial blood cultures were inconclusive and, thus, mcf-DNA sequencing was performed, yielding a positive result for two specific pathogens. Further diagnostic imaging and antibiotic regimen were guided based on these results. This case aims to highlight the clinical utility of cf-DNA sequencing in diagnosing and developing a treatment strategy for intracranial abscesses.

## Case presentation

**Figure 1:**
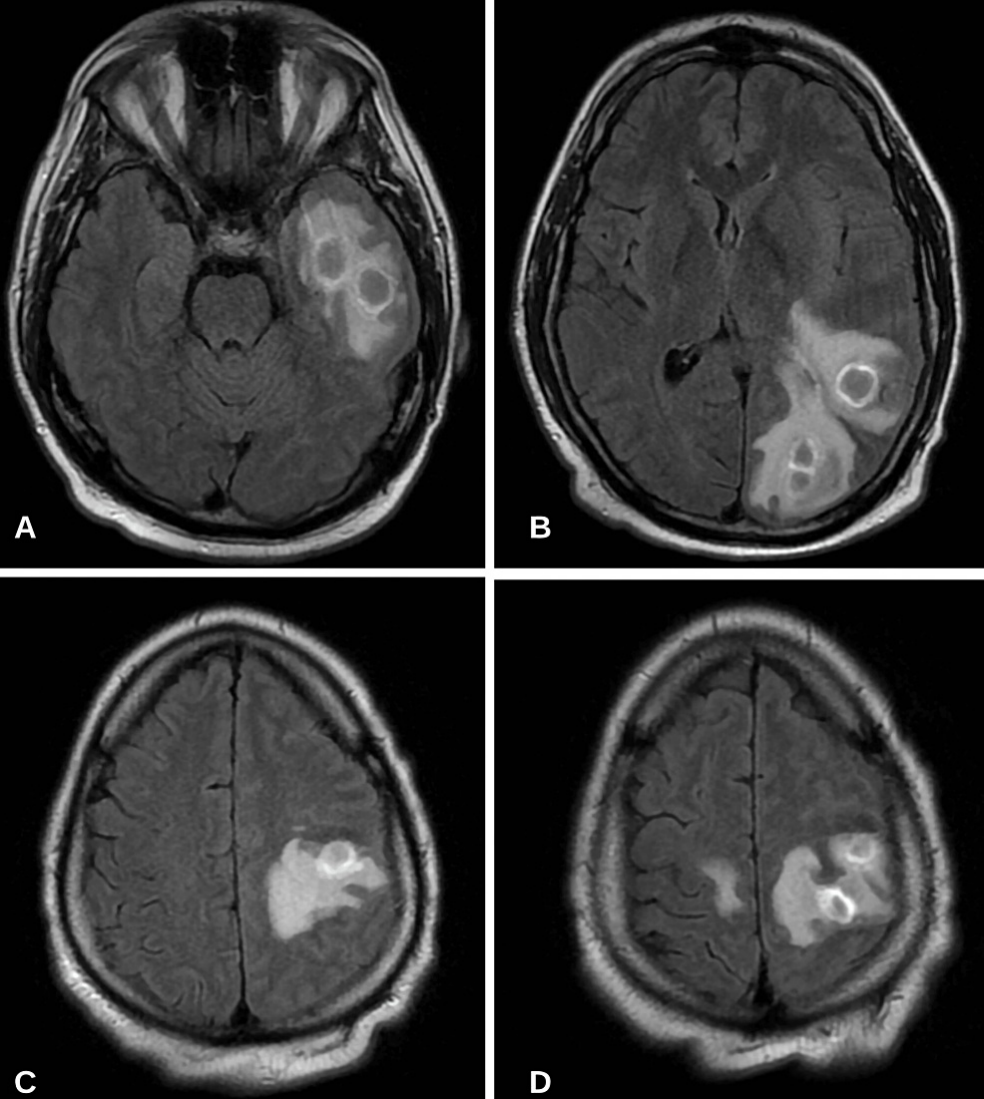
MRI brain with and without contrast T2/FLAIR sequence showing multiple focal ring-enhancing lesions and vasogenic edema (A) Two rim-enhancing lesions in the temporal lobe (largest measuring 2.1 cm); (B) One lesion at the temporo-occipital junction measuring 1.8 cm and one potentially bilobed lesion in the left occipital lobe measuring 2.3 cm; (C) One lesion in the left anterior parietal lobe measuring 1.8 cm; (D) One lesion in the posterior aspect of the left frontal lobe measuring 1.8 cm and one smaller lesion in the posterior medial aspect of the right frontal lobe. These lesions show prominent diffusion restriction and a large amount of surrounding edema. Mass effect related to the left-sided lesions including compression of the ipsilateral left lateral ventricle, third ventricle, early left uncal herniation, and up to 0.6 cm rightward midline shift. FLAIR: fluid-attenuated inversion recovery

A 34-year-old male presented to the emergency department with acute onset facial twitching and right-hand weakness. The patient denied having any history of intravenous drug abuse. Vital signs on presentation were significant for a fever of 101.1 Celsius, blood pressure of 120/75 mmHg, and heart rate of 105 beats per minute. Neurologic examination was significant for 3/5 muscle strength in the right upper extremity. Cranial nerves were intact and a Romberg test was negative. A head computerized tomography (CT) scan was completed and revealed vasogenic edema in the left hemisphere in the occipital lobe that extended towards the temporal lobe with a midline shift of 5 mm. A 1 cm lesion was also noted in the temporal lobe. Two sets of blood cultures were obtained, which later resulted in no growth. The urine and serum toxicology tests were negative. A comprehensive blood count (CBC), comprehensive metabolic profile (CMP), human immunodeficiency (HIV), hepatitis B virus (HBV), hepatitis C virus (HCV), and tuberculosis testing were obtained revealing an elevated white blood cell (WBC) count of 16,800 cells per microliter. Cryptococcal and toxoplasma antibody testing were negative. The patient was empirically started on vancomycin, cefepime, dexamethasone, and levetiracetam. MRI of the brain with and without contrast was obtained revealing multiple focal ring-enhancing lesions in the left cerebral hemisphere and a smaller lesion in the right frontal lobe (Figure 1). 

**Figure 2:**
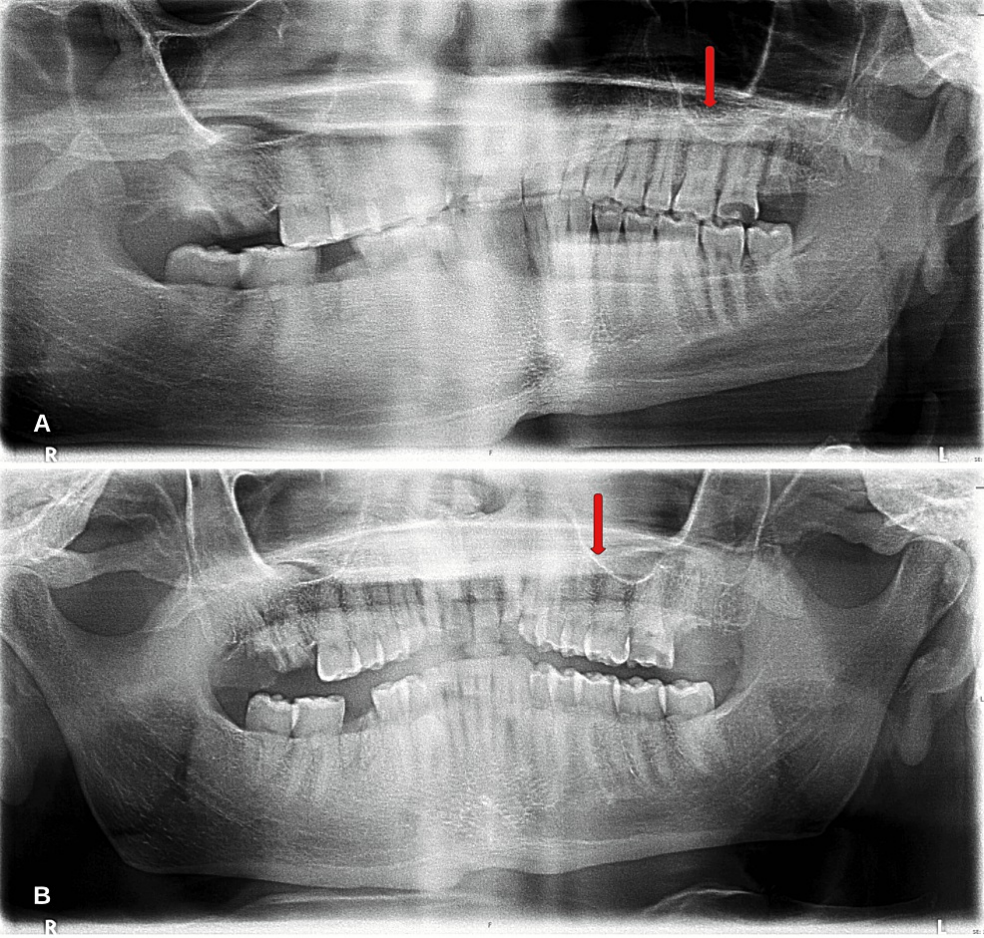
Panorex revealing poor dentition with multiple missing molars and significant dental caries in the left maxillary molars (red arrows)

A transthoracic echocardiogram was obtained revealing no vegetation. On day three of hospitalization, the patient underwent a stereotactic craniectomy for supratentorial excision and aspiration of a left occipital lesion. As a result of negative blood cultures, a next-generation sequencing test of mcf-DNA was obtained. Plasma mcf-DNA testing resulted in the growth of *Fusobacterium nucleatum* 1779 DNA molecules per microliter (MPM) (<10) and *Rothia mucilaginosa* 399 DNA MPM (<10). Antibiotics were narrowed to cefepime and metronidazole due to improved sensitivity. *Fusobacterium nucleatum* and *Rothia mucilaginosa* exist in the oral flora; therefore, a panorex was obtained revealing poor detention evident with multiple missing teeth and dental caries concerning for a dental infection (Figure 2).

**Figure 3:**
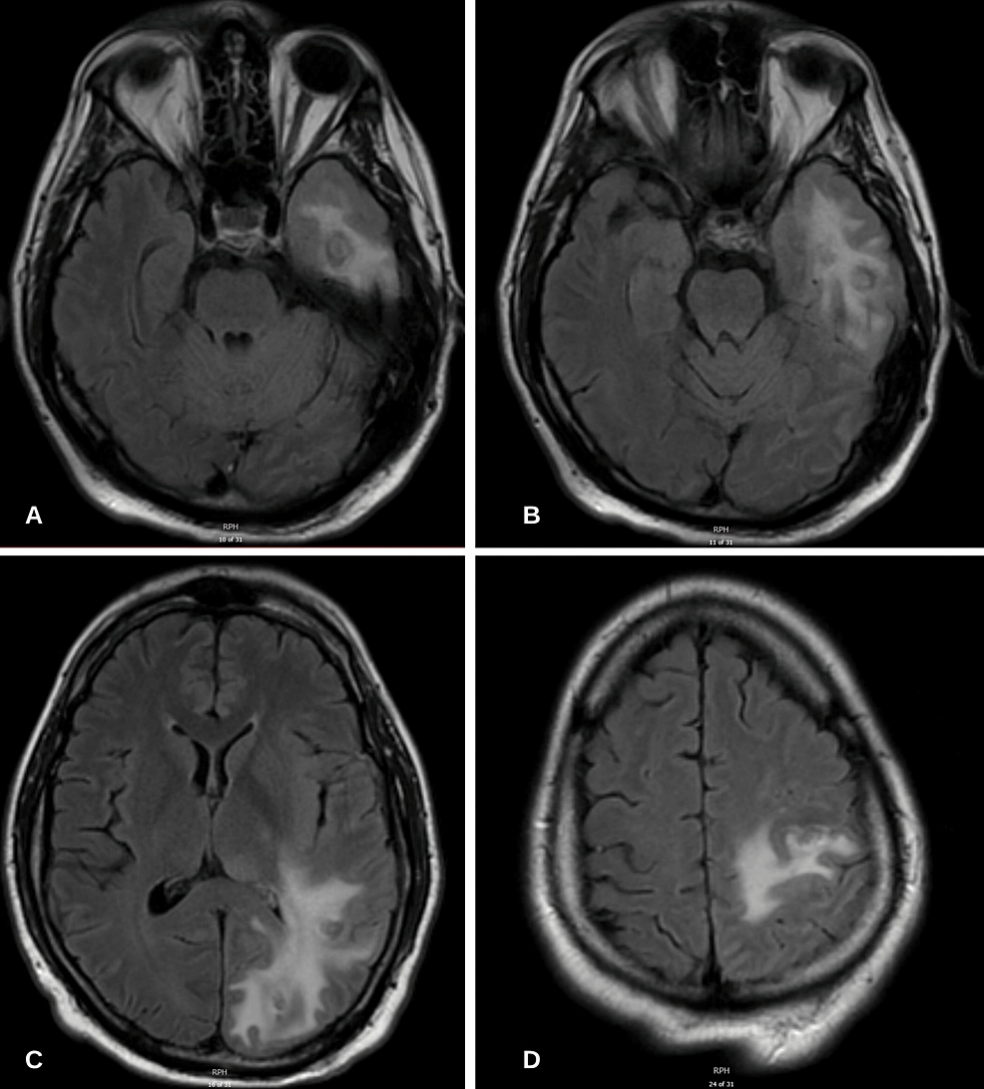
MRI brain with and without contrast T2/FLAIR sequence revealing progressive resolution of ring-enhancing lesions FLAIR: fluid-attenuated inversion recovery

On the third week of admission, a repeat MRI of the brain was completed revealing an interval decrease in the size of multiple ring-enhancing brain lesions and the associated surrounding vasogenic edema (Figure 3). The patient was continued on IV cefepime and oral (PO) metronidazole on discharge for an additional six weeks with infectious disease, and neurology follow-up. On the day of discharge, a complete neurologic assessment was completed that revealed 5/5 muscle strength in the right upper extremity and no other focal neurologic deficits.

## Discussion

**Figure 4:**
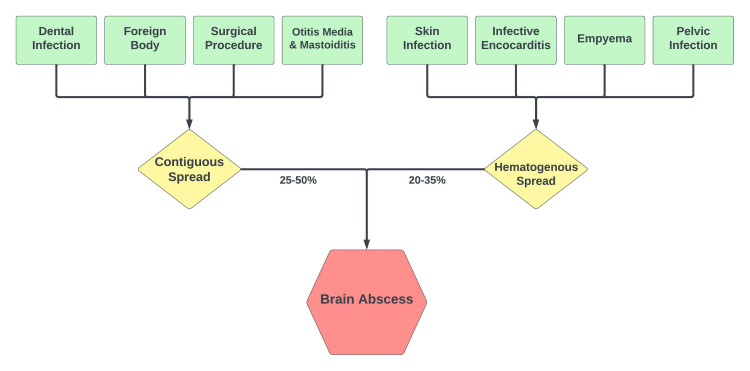
Pathogenesis of brain abscess formation Image credit: Bahadar S. Srichawla

Brain abscesses can occur due to hematogenous seeding or contiguous spread of infection. Direct spread accounts for approximately 25-50% of all cases. Hematogenous (bacteremic) seeding accounts for 20-35% of all cases [3]. The etiologies of contiguous spread include dental infection, foreign body, surgical procedure, otitis media, and mastoiditis. Etiologies of hematogenous spread include severe pulmonary infections (empyema), skin infection, and infective endocarditis. Figure 4 provides an overview of the pathogenesis of brain abscesses.

The development of brain abscesses can be attributed to four distinct stages. Early cerebritis (one to five days), late cerebritis (5-10 days), early capsule formation (11-15 days), and late capsule formation (>15 days). The most common pathogens associated with brain abscess formation are in the genus streptococcus and staphylococcus. However, a broad array of organisms are implicated in the pathogenesis of central nervous system (CNS) abscesses including fungi. However, fungal CNS abscesses are more commonly seen in immunosuppressed patients [4]. Table 1 provides an overview of the stages of brain abscess formation.

**Table 1:** The four stages of brain abscess formation

Stages	Time Course	Pathophysiology
Early Cerebritis	1-5 days	Neutrophil chemotaxis, liquefactive necrosis, and edema
Late Cerebritis	5-10 days	Macrophage and lymphocytic infiltration
Early Capsule Formation	11-15 days	Creation of abscess wall to protect normal brain parenchyma
Late Capsule Formation	> 15 days	Gliosis, granulation of abscess wall.

**Figure 5:**
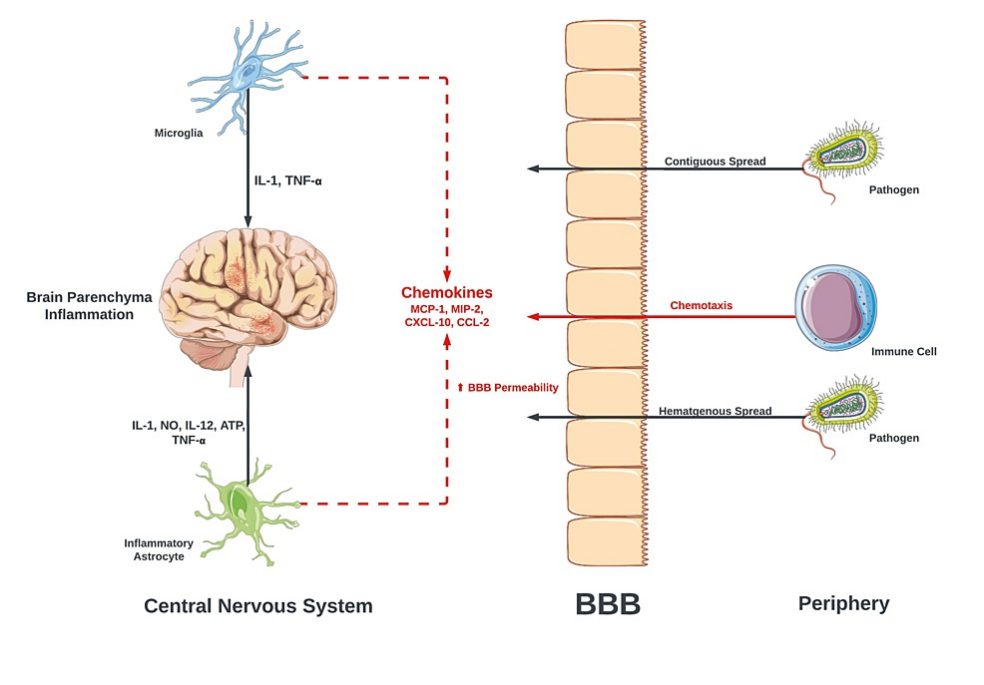
Cellular mechanisms of CNS inflammation BBB: blood-brain barrier; IL-1: interleukin-1; IL-12: interleukin-12; TNF-α: tumor necrosis factor-alpha; NO: nitric oxide; ATP: adenosine triphosphate; MCP-1: monocyte chemoattractant protein-1; MIP-2: macrophage inflammatory protein-2; CXCL-10: C-X-C motif chemokine ligand 10; CCL-2: C–C motif chemokine ligand 2 The figure was partly generated using Servier Medical Art (Servier Laboratories, Suresnes, France), licensed under a Creative Commons Attribution 3.0 unported license. Image credit: Bahadar S. Srichawla

CNS infiltration of the pathogen leads to direct brain parenchymal inflammation with the recruitment of inflammatory astrocytes and microglia. The recruitment of these inflammatory cells results in the release of cytokines, including interleukin-1 (IL-1), interleukin-12 (IL-12), and tumor necrosis alpha (TNF-α). These cytokines result in further inflammation of the brain parenchyma [5]. Inflammatory astrocytes, and microglia release chemokines that increase blood-brain barrier permeability (BBB) and lead to the recruitment of other inflammatory cells (Figure 5). Increased BBB permeability is also implicated in other CNS infections such as flaviviruses [6].

Two sets of microbial blood cultures should be obtained in patients with suspected CNS abscesses. Galactomannan and β-d-glucan should be considered to assess for a fungal infection in immunocompromised patients. MRI should include a contrast (gadolinium) study. In the early stages of capsule formation, MRI often reveals ring-enhancing lesions with areas of central necrosis. In diffusion-weighted imaging (DWI), abscesses are hyperintense, indicative of restrictive diffusion [7]. Stereotactic aspiration and brain biopsy are considered the gold standard for diagnostics; however, they may be contraindicated based on the anatomy of the lesion.

**Figure 6:**
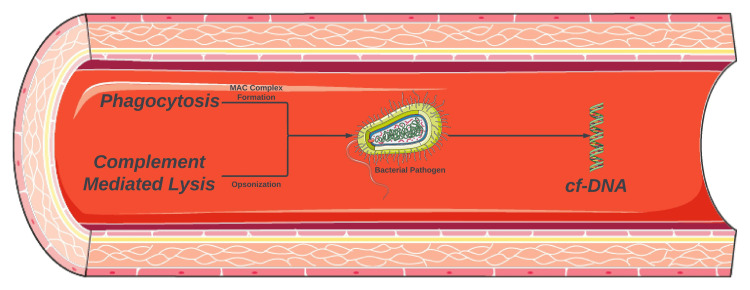
Pathogen release of cell-free DNA (cf-DNA) MAC: membrane attack complex The figure was partly generated using Servier Medical Art (Servier Laboratories, Suresnes, France), licensed under a Creative Commons Attribution 3.0 unported license. Image credit: Bahadar S. Srichawla

The location of the brain abscess may correspond to the route of infection. For example, temporal and cerebellar lesions correspond to otogenic infections. And lesions within the frontal lobe correspond to sinus infections [8]. Subcortical lesions are also observed; however, hyperintensities seen in the limbic system are more concerning for autoimmune or paraneoplastic limbic encephalitis [9]. Lesions involving the corpus callosum may occur due to an autoimmune disease process such as Susac syndrome [10]. cf-DNA is released into the bloodstream in response to apoptosis, necrosis, or immune-mediated cell death (Figure 6). These nucleic acid fragments of DNA can then be detected and reported in DNA molecules per microliter (MPM). Next-generation sequencing (NGS) provides improved sensitivity and specificity in the analysis of cf-DNA. The utilization of cf-DNA NGS has shown to be useful in diagnosing infections when numerous pathogens are in consideration [11]. In the presented case, a diagnosis of *Fusobacterium nucleatum* and *Rothia mucilaginosa* infection was determined based on mcf-DNA NGS. Both pathogens exist in the oral flora and thus a panorex was obtained revealing significant dental caries and evidence of poor dentition. This is believed to be the conduit of CNS seeding of bacteria. The cefepime and metronidazole treatment strategy were determined based on pathogen sensitivity, and clinical and radiographic improvements were noted.

Literature review

**Figure 7:**
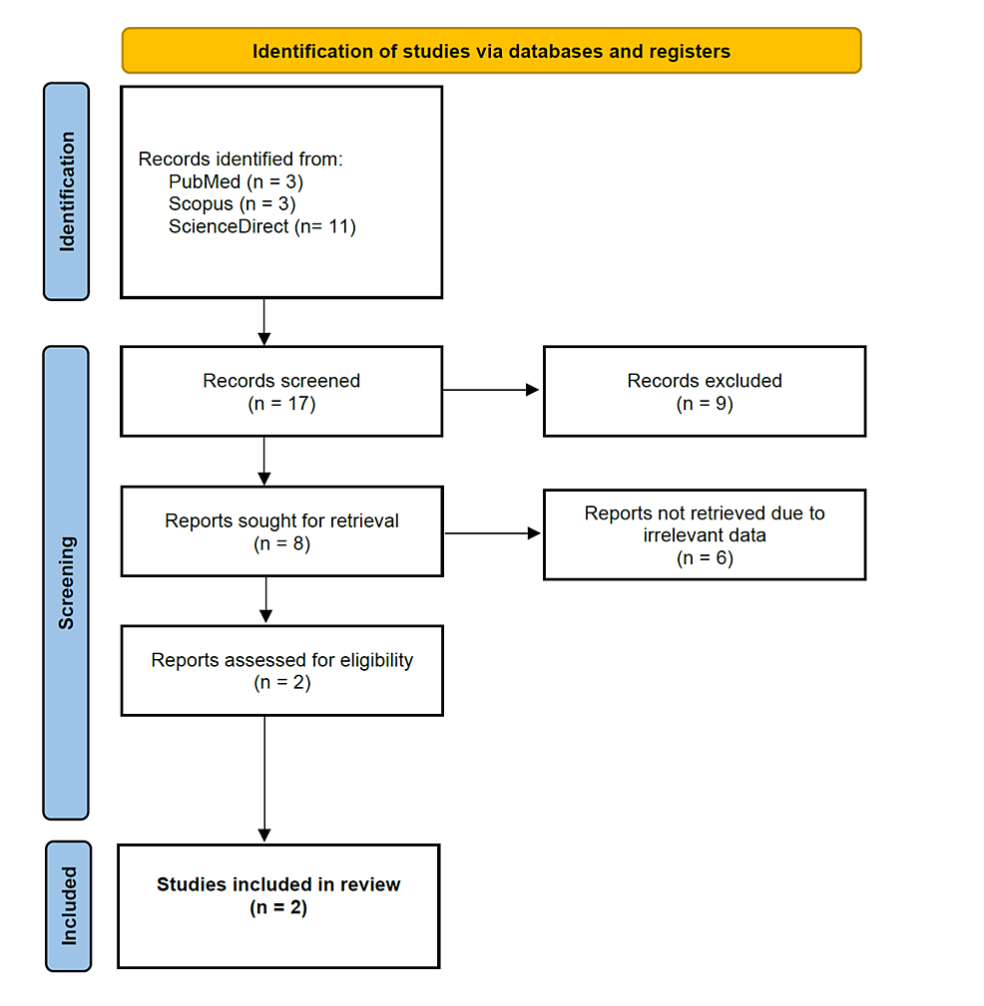
Flow diagram depicting study selection for the scoping literature review

The purpose of this scoping review is to identify cases of intracranial abscesses that were diagnosed via mcf-DNA testing. A comprehensive literature review was conducted using PubMed/PubMed Central/MEDLINE, Scopus, and ScienceDirect. The following search term was utilized: ("intracranial abscess" OR "cerebral abscess" OR "brain abscess") AND ("cfDNA" OR "cell-free DNA" OR "microbial cell-free DNA"). The search was conducted on July 15, 2022. Exclusion criteria included non-English articles and articles that did not have full-text access. Only case reports/series were included for analysis. A total of 17 records were screened and nine were excluded because they were not peer-reviewed journal case reports/series relevant to the question. Eight records were sought for retrieval and six were removed due to irrelevant data. A total of two records were assessed for eligibility and were included in the qualitative analysis. A flowchart is included for a visual depiction of the literature search (Figure 7). Only two cases were identified and are included in Table 2. The scoping review was registered on Open Science Framework (Registration DOI: 10.17605/OSF.IO/FRWV4).

**Table 2:** Cases of intracranial abscesses diagnosed with microbial cell-free DNA (cf-DNA) testing

Reference	Age	Gender	Neurological Manifestations	Pathogen	Etiology	Management
Shishido et al. (2021) [12]	43	M	Seizures	*Aspergillus fumigatus* (invasive aspergillosis)	Liver transplant recipient	Voriconazole
Pareek et al. (2021) [13]	9	F	Vomiting, gait ataxia, facial and ocular motor nerve palsies	Cladophialophora bantiana	Exposure to contaminated soil	Voriconazole, flucytosine, and intraventricular amphotericin B.

Shishido et al. (2021) reported a case of a 43-year-old male presenting with nausea, vomiting, and chills. The patient was diagnosed with fulminant liver failure from hepatitis A. He was found to have generalized tonic-clonic (GTC) seizures. An MRI of the brain revealed multiple parenchymal lesions consistent with brain abscesses. An mcf-DNA assay was positive for *Aspergillus fumigatus*, and the patient was managed with voriconazole. Repeat MRI after six weeks of therapy showed an interval decrease in the size of brain lesions. And an MRI four months later revealed near resolution of the lesions [12].

Pareek et al. (2021) reported a case of a nine-year-old female presenting with occipital headache, gait ataxia, and visual disturbance. The patient had 20/40 visual acuity in the left eye, right-sided dysmetria, and multiple cranial nerve deficits. Throughout multiple prolonged hospital courses, the patient had MRI findings consistent with multiple lesions involving the pons and cerebellum. Plasma mcf-DNA sequencing revealed colonization with *Cladophialophora bantiana*. The patient was managed with voriconazole, flucytosine, and intraventricular amphotericin B [13].

A total of two cases are identified in this scoping review. Both cases highlight the key utility of cf-DNA sequencing in identifying the underlying pathogen and subsequent treatment strategy. As the availability of cf-DNA NGS becomes more widely available, a greater number of reported cases of CNS infection diagnosed via cf-DNA NGS can be expected. A limitation of the current review includes the utilization of only three databases in the search strategy.

## Conclusions

CNS abscesses are of significant morbidity and mortality. The case of a 34-year-old male with multiple intracranial abscesses is reported. Standard diagnostic microbial cultures did not yield diagnostic value. NGS of mcf-DNA was used to determine two bacterial pathogens *Fusobacterium nucleatum* and *Rothia mucilaginosa*. Both pathogens exist as part of the oral flora, and further diagnostic imaging with a panorex revealed multiple missing molars and dental caries consistent with a dental infection. Treatment included cefepime and metronidazole. Repeat MRI revealed an improvement in previously identified ring-enhancing lesions. The microbial NGS of cf-DNA constituted a cardinal role not only in diagnosing but also in developing a treatment strategy for a life-threatening infection. A scoping literature review of intracranial abscesses diagnosed by cf-DNA sequencing is included.
